# 2s-DAS: Two-Stream Diffusion with Multi-Modal Fusion for Temporal Action Segmentation

**DOI:** 10.3390/jimaging12060237

**Published:** 2026-05-28

**Authors:** Ce Li, Xuli Guo, Ruijie Wang, Kaipan Zhao, Linlin Yang, Fang Wan

**Affiliations:** 1 School of Artificial Intelligence, China University of Mining and Technology-Beijing, Beijing 100083, China; 2 State Key Laboratory of Media Convergence and Communication, Communication University of China, Beijing 100024, China; 3 University of Chinese Academy of Sciences, Beijing 100049, China

**Keywords:** temporal action segmentation, diffusion model, multi-modal fusion, spatial-temporal representation

## Abstract

Human temporal action segmentation (TAS) is a fundamental video understanding task aimed at partitioning untrimmed videos into semantically coherent action segments. While temporal convolutional networks and transformers have significantly improved frame representation and temporal modeling, existing methods are still constrained by two critical limitations: the dependence on single-modal inputs and the inefficiency of iterative, frame-wise sequential modeling. To address these gaps, we propose 2s-DAS: a novel two-stream diffusion-based framework for action segmentation characterized by three key contributions. First, we introduce a multi-modal frame representation that integrates optical flow with Br-Prompt RGB features, thereby capturing richer spatial-temporal context and enhancing feature representation. Second, we leverage a diffusion model to perform sequence segmentation, utilizing importance sampling to prioritize key frames for segment-level temporal modeling. Concurrently, a refinement mechanism based on iterative decoding denoising is introduced to ensure fine-grained action prediction. Third, we design a two-stream fusion mechanism that processes the streams of RGB with text and optical flow separately and integrates multi-modal information by a late fusion strategy to explicitly reduce oversegmentation. Evaluation experiments on GTEA, 50Salads, and Breakfast datasets show that our 2s-DAS significantly outperforms state-of-the-art methods, setting new benchmarks while effectively addressing the over-segmentation issue.

## 1. Introduction

Temporal action segmentation (TAS) is an important research topic in the fields of human-centric video analysis and understanding, aiming to decompose continuous video streams into distinct action segments and assign precise labels to each frame. It finds critical applications in human behavior analysis [1,2,3], human–computer interaction systems [4] and beyond.

Recent methods in action segmentation typically extract feature representations from videos using a pretrained model, then refine these features and predictions through multi-level iterative strategies within a segmentation framework. Despite advances brought by temporal convolutional networks (e.g., MS-TCN [5]) and transformers (e.g., ASFormer [6], UVAST [7]), existing methods still suffer from three key limitations: (1) over-reliance on single-modal (RGB) inputs, (2) high computational cost in modeling long-range dependencies, and (3) over-segmentation artifacts caused by frame-wise iterative refinement. While recent diffusion models [8,9] have recently shown promise for temporal modeling, they remain uni-modal and lack explicit mechanisms for precise segment boundary refinement.

Inspired by the advances in multi-modal learning from audio-visual speech recognition research (e.g., MCFM [10]), we observe that complementary multi-modal fusion holds great potential to address these gaps. Fusing RGB with optical flow, text, or audio [11,12,13] enriches contextual representation and improves robustness. However, to the best of our knowledge, no prior work has successfully integrated diffusion models with multi-modal fusion in the context of TAS. To bridge this gap, we propose a novel two-stream diffusion framework with multi-modal fusion named 2s-DAS for TAS.

**Figure 1:**
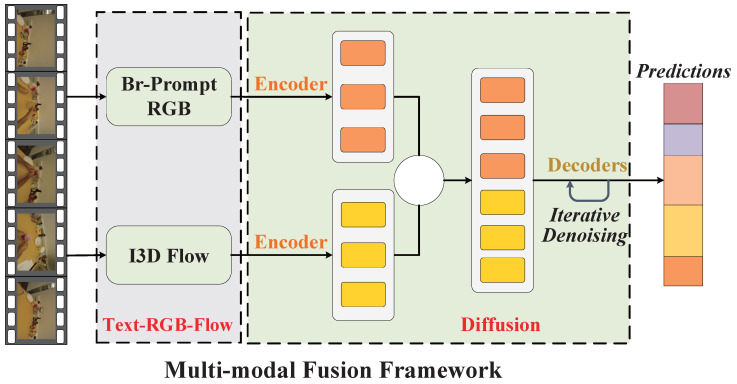
The framework of two-stream diffusion with multi-modal fusion (2s-DAS) for TAS.

As shown in Figure 1, the proposed 2s-DAS framework introduces a two-stream fusion mechanism that incorporates Br-Prompt RGB features [14] and I3D optical flow features [15], which contain richer scene cues within each frame and thereby enhance the feature representation. In our two-stream design, one stream processes RGB-with-text features (Br-Prompt RGB) to capture static semantic cues among actions, while the other handles optical flow features (I3D flow) to model dynamic motion patterns. We adopt a late-fusion strategy that combines the two streams via weighted integration, allowing for flexible modality fusion. For temporal modeling, we employ diffusion models in both streams, which extract modality-specific feature information through encoders and use an importance sampling strategy to select key frame features for segment-level temporal modeling. These features are then fed into the decoder for iterative denoising, ultimately producing the original action label sequence. We evaluate our method on three challenging benchmark datasets(GTEA, 50Salads, and Breakfast), and the results demonstrate that our approach significantly outperforms baseline methods and achieves superior performance compared to current state-of-the-art approaches. In summary, our contributions are as follows:

- We design a novel multi-modal fusion framework named 2s-DAS to fuse Br-Prompt RGB and I3D Flow, representing Text-RGB-Flow fusion, and achieve the SOTA results on benchmarks, which is a successful demonstration of the “diffusion + multi-modal learning” for the temporal action segmentation task. It is noted that our framework has generality and plug-in convenience across different backbones and multiple modalities.
- We introduce a diffusion-based backbone equipped with an importance sampling strategy via zero-shot random selection and a multi-level decoder. This design captures the prior distribution of action sequences through multi-step denoising, akin to iterative optimization, enabling more precise adjustments to segment predictions.
- Extensive experiments on three challenging benchmarks demonstrate that 2s-DAS achieves competitive or superior performance compared to state-of-the-art models.

## 2. Related Works

### 2.1. Temporal Action Segmentation

Temporal action segmentation has evolved from early sliding window and Markov models to advanced neural architectures. Temporal Convolutional Networks (TCNs) model local and long-range temporal patterns by stacking feed-forward convolutional layers, offering stronger parallel computing capabilities than early methods. Lea et al. [16] first introduced the Encoder-Decoder TCN (ED-TCN) for temporal action segmentation. This framework was later enhanced by Ding et al. [17], who incorporated an LSTM decoder to strengthen temporal dependency modeling. However, since temporal downsampling in early architectures often degraded fine-grained boundary details, Farha et al. [5] proposed the Multi-Stage TCN (MS-TCN). By cascading multiple stages with progressive dilated convolutions, MS-TCN effectively enlarges the receptive field while maintaining full temporal resolution, thereby significantly alleviating over-segmentation. This architecture was further optimized by Li et al. [18] through dual dilated convolutions and parameter-sharing strategies. More recently, Transformers have been widely adopted to enhance global context modeling. Yi et al. [6] proposed ASFormer, which combines dilated convolutions and self-attention in the encoder, utilizing cross-attention in multi-stage decoders for iterative refinement. Building upon this, subsequent studies introduced autoregressive decoding for transcript prediction [7], multi-level dilated Transformers (MSDTN) [19] for simultaneously modeling local and global temporal relations, and sparse attention mechanisms (LT-Context) to capture more complete long-range contexts [20]. Finally, to address the data-hungry nature of Transformers and the over-segmentation issues of TCNs, hybrid architectures like TCTr [21] were developed to seamlessly integrate convolutional stages with Transformer encoders, achieving a balance between computational complexity and segmentation performance. Furthermore, the significance of robust temporal feature integration and sequential modeling is also evidenced in the development of automated pipelines for specific target tracking and classification in other complex video domains, such as sperm analysis in microscopy videos [22,23].

Recently, diffusion models have revolutionized the broader field of video analysis, showing immense promise for complex temporal tasks. Beyond static image generation, milestone works in video generation, such as Video LDM [24] and Make-A-Video [25], have demonstrated the exceptional capability of diffusion architectures in modeling high-dimensional spatial-temporal data distributions. Furthermore, extending these generative priors to discriminative video understanding, recent methods like DiffTAD [26] have successfully applied the iterative denoising paradigm to temporal action detection, proving its efficacy in precise boundary localization. In the specific context of temporal action segmentation, the diffusion paradigm provides inherent optimization for sequence alignment, with noise perturbation significantly enhancing the model’s robustness against boundary ambiguity. Liu et al. [9] pioneered diffusion for action segmentation by reframing it as action sequence generation, demonstrating superior handling of long videos. Building on this potential, we adopt diffusion modeling as our backbone framework.

### 2.2. Multi-Modal Fusion

Multi-modal learning has gained significant traction for enhancing model performance across vision tasks [11,12,13]. For instance, recent advances in medical imaging have demonstrated that integrating deep image features with complementary clinical data via multiple-instance learning can effectively overcome the inherent limitations of single-modal representations [27]. In video understanding, integrating complementary modalities (e.g., text, audio, optical flow) enriches feature representations and improves robustness [12,28]. For action segmentation specifically, early methods fused RGB and optical flow via simple concatenation [15]. Recent advances employ sophisticated fusion strategies, such as Ishihara et al.’s mid-fusion module (MCFM) combining pose and RGB data [10]. Optical flow remains particularly valuable for capturing temporal dynamics in action sequences [29]. Our work extends this direction by designing a dual-stream diffusion framework to leverage optical flow’s motion cues alongside RGB features.

## 3. Methodology

**Figure 2:**
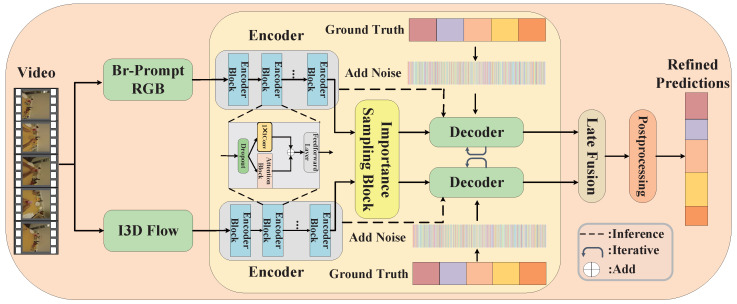
The framework of 2s-Das. The input video is divided into frames, and the corresponding Br-Prompt RGB and I3D optical flow features are extracted by two feature extraction models, i.e., Br-Prompt and I3DNet. These two streams of features are then input into the encoders to extract higher-level temporal feature information. Then, for temporal segment-level modeling of each stream of feature sequence, the diffusion module is designed with the importance sampling block to select key frames’ information as the input of the decoder, along with the action sequence with added noise. Finally, the denoised action sequences from two decoders are input into the late fusion layer and post-processing for fusion of multi-modal information and refining the prediction outputs.

In this section, we will provide a detailed introduction to the overall structure of 2s-DAS. Section 3.1 elaborates on the design of 2s-DAS, modality fusion, and the corresponding modality feature extraction methods. Section 3.2 introduces the design of the diffusion model based on the encoder–decoder structure. The overall structure of 2s-DAS is shown in Figure 2.

### 3.1. 2s-DAS Framework

**Pipeline**. In this work, we propose the 2s-DAS framework to address the temporal action segmentation task. Our 2s-DAS employs two diffusion models with encoder-decoder structures, each receiving RGB and FLOW feature sequences, respectively. Given pre-extracted frame-by-frame video feature sequences $F_{R}$ and $F_{F}$, of size T×d, where *T* is the length of the video sequence and *d* is the dimension of the input features, the feature sequence is input into the encoder to extract higher-level feature representations. Subsequently, the encoder outputs a predicted label sequence $Y_{\mathrm{out}}$ of size B×C×T, where *B* is the batch size, *C* is the number of action categories, and *T* remains the length of the video sequence. $Y_{\mathrm{out}}$ is used to compute the loss function for the encoder, updating the learning parameters within the encoder. Additionally, the encoder generates a higher-level feature representation $F^{\prime }$, which is input into the decoder as conditional information combined with noise data to help guide the decoder in recovering the original action label sequence from the noise. The encoder also provides feature map mappings from different layers as additional inputs to the decoder, enhancing its understanding and modeling capabilities of the video content. The decoder applies different importance sampling strategies (Section 3.2) to the input features, and its inputs also include the diffusion step n∈{1,2,…,N} and the action label sequence $Y_{n}$ with added noise. The decoder will denoise $Y_{n}$ based on this input information to generate the denoised sequence $Y_{n-1}$ at the previous time step n−1. Through multiple iterations of this process, the prediction result *P* that is close to the original action label sequence can ultimately be obtained. Finally, the prediction information $P_{fusion}$ from the two streams is fused through a weighted combination to achieve late fusion. The fusion formula is as follows:

(1) $$P_{fusion}=\mu_{R}P_{R}+\mu_{F}P_{F},\;\;\left\{\mu_{R}+\mu_{F}=1\right\}$$

The fused prediction result is denoted as $P_{fusion}$, where $P_{R}$ and $P_{F}$ correspond to the output results of the RGB stream and the optical flow stream, respectively. $\mu_{R}$ and $\mu_{F}$ are the weights for each stream, with values ranging between 0 and 1. Depending on the dataset, we set these weight parameters to different values. It is important to emphasize that our choice of a weighted late-fusion strategy over more complex early or mid-fusion fusion modules is fundamentally structural. Since RGB and optical flow possess inherently different feature distributions and temporal dynamics, coupling them early in the iterative diffusion denoising process causes gradient interference and degrades the learning of modality-specific priors. As validated in our ablation studies in Section 4.3, allowing two independent diffusion streams to fully recover their sequence priors before a late fusion yields the most robust multi-modal synergy. Finally, post-processing strategies such as purge, median filtering, or mode filtering are applied to the fused prediction results to further refine the outcomes.

**Feature extraction.** We utilize the pre-trained I3D [15] model to extract I3D FLOW features, where the feature dimension *d* is set to 1024. For RGB feature extraction on the GTEA and 50Salads datasets, we replace the standard I3D RGB features with those generated by the pre-trained image encoder from the Br-Prompt [14] framework. Regarding the prompting details, the original Br-Prompt framework aligns video clips with a unique “three-plus-one-level” textual prompt structure via contrastive learning. This structure dynamically generates text templates integrating statistical, ordinal, semantic, and integrated prompts to capture temporal logic. It is crucial to note that in our 2s-DAS framework, we do not manually design or fine-tune these textual prompts during our training phase. Instead, we directly extract the 768-dimensional (d=768) frame-wise RGB features generated by the frozen Br-Prompt vision encoder. By doing so, our extracted features inherently inherit the rich ordinal and semantic contextual awareness learned during Br-Prompt’s pretraining, without requiring any additional prompting overhead in our pipeline.

### 3.2. Diffusion Module

When applied to action segmentation tasks, diffusion models primarily consist of two phases: the forward process and the reverse process. Since both the RGB and optical flow streams employ an identical diffusion architecture, we introduce the detailed mathematical formulation by taking a single stream as a generic example and omitting the stream-specific subscripts for clarity. To effectively bridge the gap between the continuous diffusion paradigm and the discrete categorical label space, we first represent the action label sequence in a continuous logit space $Y_{0}\in R^{C\times T}$ via one-hot encoding, where *C* is the number of action categories and *T* is the sequence length. This ensures that the model can perform iterative refinement within a stable probability space. Under this formulation, the forward process involves gradually adding Gaussian noise to the continuous representation $Y_{0}$, making it increasingly random until it becomes a pure noise sequence $Y_{N}$, which almost completely loses the original action information. This diffusion process can be described by Equation (2):

(2) $$Y_{n}=\sqrt{\sigma_{n}}\;Y_{0}+\sqrt{1-\sigma_{n}}\;\theta_{n},\;n\in \left\{1,2,\ldots ,N\right\}$$

where $Y_{0}$ is the original action sequence label, $\theta_{n}$ represents random noise used to simulate the uncertainty and ambiguity in the action sequence at step *n*, and $\sigma_{n}$ controls the degree of noise added at each step. The reverse process primarily starts from the pure noise action sequence $Y_{N}$ and recovers the original action label $Y_{0}$ by gradually removing noise. The denoising process at each step *n* can be described by Equation (3):

(3) $$Y_{n-1}=\sqrt{\sigma_{n-1}}P_{n}+\sqrt{1-\sigma_{n-1}-\omega_{n}^{2}}\frac{Y_{n}-\sqrt{\sigma_{n}}P_{n}}{\sqrt{1-\sigma_{n}}}+\omega_{n}\theta_{n}\;n\in \left\{1,2,\ldots ,N\right\}$$

where $P_{n}$ is the denoised sequence representing the estimated original sequence $Y_{0}$, which is obtained by inputting the noisy sequence $Y_{n}$ into the decoder at step *n*. The variance parameter $\omega_{n}$ controls the stochasticity of the reverse trajectory. By iteratively applying Equation (3) until $Y_{0}$ is obtained, an argmax operation is finally applied along the class dimension to map the continuous logits back into discrete categorical action labels.

During the training phase, we randomly select a diffusion step n∈{1,2,…,N} for each batch. We apply Gaussian noise $\theta_{n}∼N\left(0,I\right)$ to the ground-truth label sequence $Y_{0}$, resulting in the noisy input $Y_{n}$. The decoder is then trained to estimate the original sequence $Y_{0}$ from this corrupted version. In the inference phase, the model starts with a fully noisy sequence $Y_{N}$. It iteratively refines this sequence using the DDIM denoising procedure. This process progressively generates cleaner outputs until the final prediction *P* is obtained. The importance sampling strategy is utilized exclusively during the training phase. By selectively masking input features, the framework is incentivized to capture the prior distribution of human actions and maintain structural consistency under information constraints. During inference, this strategy is deactivated to allow the model to leverage unabridged input features, thereby achieving superior boundary refinement and segmentation accuracy. Therefore, it can denoise the pure noise sequence $Y_{N}$ based solely on the features *F* generated by the encoder. The training procedure of our model is as follows:

(4) $$P_{n}=D_{ec}\left(Y_{n},n,\left(E_{nc}\left(F\right)⊙M\right)\right)$$

where $P_{n}$ is the estimated original sequence at step *n*, $D_{ec}$ denotes the decoder, $E_{nc}$ represents the encoder, ⊙ indicates element-wise multiplication, and M is the mask generated by the sampling strategy in the importance sampling module.

**Encoder.** The diffusion module consists of an encoder, a decoder, and an importance sampling block. The encoder is composed of *L* encoder blocks, each built around a dilated convolution, a dilated attention mechanism, and a feed-forward layer, adapted from ASFormer [6]. The input features are first processed through the dilated convolution to extract local features, followed by the dilated attention to model the global context of the features. Specifically, in the dilated attention, the receptive field of the *i*-th block is restricted to a local window of size $w=2^{i}$, achieving progressive temporal modeling from local to global by dynamically adjusting the dilation rate. After computation in each block, the output is added to the input features via a residual connection before being passed to the next encoder block. Subsequently, the features are fed into the feed-forward network to generate new feature representations, which are then mapped into the class space to produce the initial action segmentation prediction. **Decoder.** The decoder shares similar convolutional layers and attention mechanisms with the encoder. The only difference is that it requires logical transformations of the time steps. It also employs cross-attention mechanisms to compute the influence of attention weights among multiple inputs within the attention module. The decoder receives the current features and the noisy action sequence, and iteratively generates a precise action sequence via the reverse denoising process defined in Equation (3).

**Importance sampling.** Before feeding the refined features $F^{\prime }$ into the decoder, we use an importance sampling strategy to filter the information that needs to be passed to the decoder, thereby guiding the model to learn the action prior distribution and predict results. During the training phase, we follow the method in [9] and randomly select among the following three strategies: full sampling, boundary sampling, and random action sampling. Zero sampling is excluded because it relies solely on prior modeling—predicting based on the initial positions of video frames, which cannot dynamically adjust actions during decoding and does not align with our video action segmentation task goals. Specifically: **(a) Full sampling.** Inputs all features into the decoder, helping the model directly learn a discriminative mapping from features to action classes. **(b) Zero sampling.** Does not pass any features, directly using the initial action positions for prediction (not applicable to action segmentation tasks). **(c) Boundary sampling.** Removes features near action boundaries, forcing the model to rely on contextual information to refine boundary predictions. **(d) Random action sampling.** Randomly removes features of a specific action class, enabling the model to infer missing regions through contextual information from other action classes.The design of our importance sampling module is rooted in the need to provide the decoder with diverse contextual perturbations, thereby compelling the model to learn a robust generative prior of action sequences. While specific strategies like boundary sampling emphasize the refinement of action transitions, relying exclusively on a single structured sampling method can lead to localized over-fitting. We propose a Random Selection strategy that dynamically chooses among the defined sampling methods during each training iteration. This approach serves as a form of stochastic regularization (analogous to Dropout), preventing the model from becoming overly dependent on specific temporal cues. By encountering a wide variety of masked and unmasked feature combinations, the decoder is forced to model the global temporal logic and holistic action patterns. As demonstrated in our ablation study (in Section 4.3), this randomized strategy yields superior performance over any individual structured sampling method, effectively enhancing the model’s ability to handle both boundary ambiguity and long-range sequence alignment.

### 3.3. Loss Function

We compare the denoised prediction sequence *P* obtained from the decoder with the original action value labels $Y_{0}$ to calculate the corresponding loss values. The loss function in this paper consists of three parts: cross-entropy classification loss $L_{\mathrm{ce}}$, smoothing loss $L_{\mathrm{sm}}$ [5], and boundary alignment loss $L_{b}$.

The cross-entropy classification loss $L_{\mathrm{ce}}^{n}$ uses the minimization of negative log-likelihood loss, and its formula is shown in Equation (5):

(5) $$L_{\mathrm{ce}}=-\frac{1}{TC}\sum_{t=1}^{T}\sum_{c=1}^{C}Y_{0,t,c}\log\left({\hat{Y}}_{t,c}\right)$$

where *t* denotes the video sequence time step index, *c* denotes the action class, $Y_{0,t,c}$ represents the original ground-truth label of the *t*-th frame for class *c*, and ${\hat{Y}}_{t,c}$ is the corresponding predicted probability obtained by applying a Softmax function over the predicted logits. The smoothing loss $L_{\mathrm{sm}}$ promotes the temporal smoothness of the model’s output by calculating the mean squared error of the frame-by-frame probabilities, and its formula is shown in Equation (6):

(6) $$L_{\mathrm{sm}}=\frac{1}{TC}\sum_{t=1}^{T}\sum_{c=1}^{C}{\left({\hat{Y}}_{t,c}-{\hat{Y}}_{t-1,c}\right)}^{2}$$

The boundary alignment loss $L_{b}$ ensures that the boundaries detected in the denoised sequence $P_{n}$ align with the boundaries in the true sequence $Y_{0}$. We use a binary cross-entropy loss to calculate the boundary alignment loss between the ground-truth boundary sequence $B_{gt}$ derived from $Y_{0}$ and the predicted boundary probabilities $B_{p}$ derived from the denoised sequence $P_{n}$. The final loss function is composed as shown in Equation (7):

(7) $$L=L_{\mathrm{ce}}^{enc}+L_{\mathrm{sm}}^{enc}+L_{\mathrm{ce}}^{dec}+L_{\mathrm{sm}}^{dec}+L_{b}^{dec}$$

For 2s-DAS, the loss function for each stream is composed of L, which we define as the RGB stream loss function $L_{R}$ and the optical flow stream loss function $L_{F}$. To allow the model to simultaneously optimize the performance of both streams during training, we connect the loss functions of the two streams. This enables different streams to learn from each other through gradient propagation and relatively reduces the complexity of training, improving the stability and convergence rate. Therefore, the total loss function of the model $L_{\mathrm{total}}$ is as follows:


(8) $$L_{\mathrm{total}}=L_{R}+L_{F}$$

### 3.4. Implementation and Training Details

The overall structure of the 2s-DAS model includes two encoders and two decoders. Both the encoders and decoders are improved based on the ASFormer [6] model, and by reducing iterative calculations, the decoders in this paper significantly reduce computational costs. Each encoder consists of 10 encoder blocks, with an input dimension of 64 for the 50Salads and GTEA datasets, and 256 for the Breakfast dataset. Each decoder includes 8 decoder blocks, with dimensions of 24 for the 50Salads and GTEA datasets, and 128 for the Breakfast dataset. The convolution kernel size for the GTEA and Breakfast datasets is 5, while for the 50Salads dataset, the convolution kernel size in the decoder is set to 7. Regarding the training protocols, both the Br-Prompt and I3D feature extractors are pre-trained on Kinetics-400 and kept strictly frozen during the training of our framework. The spatial resolution of the input video frames is set to 224 × 224, and features are extracted offline to ensure computational efficiency. For the optimization process, we employ the Adam optimizer with a uniform batch size of 4 across all experiments. For the 50Salads and GTEA datasets, the learning rate is 0.0005, while for the Breakfast dataset, it is 0.0001. The total number of training epochs is set to 10,000 for the GTEA dataset, 5000 for 50Salads, and 1000 for the Breakfast dataset. The fusion weights $\mu_{R}$ and $\mu_{F}$ are determined through grid search on the validation set of each fold. We implemented the 2s-DAS structure using PyTorch 1.10, and all experiments were conducted on NVIDIA Tesla V100. Similar to [9], the total number of diffusion steps is set to 1000. During inference, we adopt the accelerated sampling strategy with a step size of 25 [30]. The multi-modal late-fusion weights and other hyperparameters are determined through an experimental search exclusively on the training folds. No information from the test folds is utilized during the model selection or hyperparameter tuning process.

## 4. Experiments and Results

### 4.1. Datasets

**GTEA** [31] dataset comprises 28 egocentric videos with a frame rate of 15 FPS, focusing on daily kitchen activities. These videos involve 11 action classes, with an average of 20 action instances per video. Each video has an average duration of half a minute. **50Salads** [32] dataset contains 50 videos of salad making with a frame rate of 30 FPS. The videos are recorded from a top-down angle. They were captured by 25 individuals, each making two salad-related preparation videos. All videos cover 17 different actions, with an average of 20 action instances per video. **Breakfast** [33] dataset is the largest and most challenging among the three datasets, containing 1712 third-person 15 FPS breakfast preparation activity videos. All 10 different types of breakfast activities were performed by 52 different individuals in 18 different kitchens. The dataset covers 48 different actions, with an average of 6 action instances per video. For evaluation, we conduct four-fold cross-validation on the GTEA and Breakfast datasets and report the average results. For the 50Salads dataset, we conduct five-fold cross-validation. For evaluation, we strictly follow the standard K-fold cross-validation protocol as established in previous works (e.g., MS-TCN [5]). Specifically, we conduct four-fold cross-validation on the GTEA and Breakfast datasets, and five-fold cross-validation on the 50Salads dataset, reporting the average results across all folds. Importantly, these standard data splits are strictly subject-independent, ensuring that the subjects in the test set never appear in the training sets. This rigorous separation guarantees the generalization capability of the proposed model and explicitly rules out the possibility of data leakage.

**Metrics for evaluation.** To evaluate the performance, we use three evaluation metrics following previous works: Frame Accuracy (Acc), Segmental Edit Score (Edit), and Segmental Overlap F1 Score. Frame accuracy reflects the ratio of frames correctly predicted by the model to the total number of frames. It is the most commonly used evaluation metric in action segmentation. The Segmental Edit Score is used to address over-segmentation errors. It penalizes excessive segmentation errors during operations. The Segmental Overlap F1 Score is utilized to assess the quality of action segmentation. It is calculated at different thresholds of 0.1, 0.25, and 0.5, denoted as F1@{10,25,50}.

### 4.2. Comparison with SOTA

We compare the proposed 2s-DAS multi-modal feature fusion method with existing state-of-the-art methods on the 50Salads, GTEA, and Breakfast datasets, as shown in Table 1. For the 50Salads and GTEA datasets, we fuse Br-Prompt RGB and I3D FLOW features. Compared to the baseline method DiffAct, on the 50Salads dataset, the edit score improves by 3.0, frame accuracy (Acc) increases by 0.6, and F1@{10,25,50} improve by 2.2, 2.5, and 2.5, respectively; on the GTEA dataset, the edit score improves by 3.8, and F1@{10,25,50} improve by 2.3, 2.5, and 1.4, respectively. Compared with other state-of-the-art methods, our evaluation results also achieve a leading performance.

**Figure 3:**
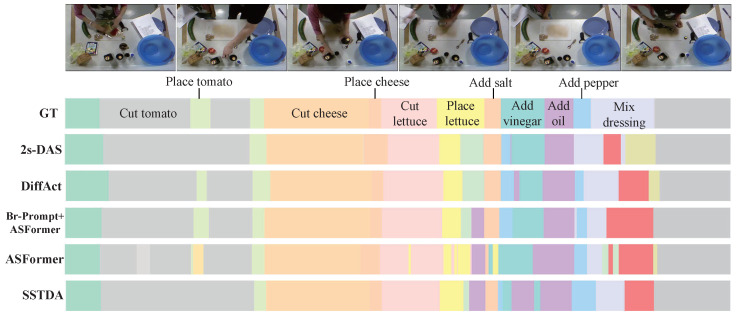
Qualitative results for action segmentation task on the 50Salads dataset, the relevant behaviors were annotated on color blocks and compared with other methods.

Regarding feature extraction, we use Br-Prompt RGB for GTEA and 50Salads, as it handles egocentric and top-down activities well. For the Breakfast dataset, however, we switch to standard I3D RGB features. This choice is based on two practical considerations. First, Breakfast consists of unconstrained third-person videos across diverse environments. We empirically observed a domain gap here, where Br-Prompt struggles to extract effective representations. Second, and more importantly, standard I3D features are the universally accepted baseline for the Breakfast dataset in recent works (e.g., DiffAct, ASFormer). By aligning our feature inputs with these methods, we ensure a strictly fair comparison, confirming that the performance gains come from the proposed 2s-DAS architecture itself rather than a stronger feature extractor. The results show that compared to the baseline DiffAct [9], the edit score of the 2s-DAS model improves by 1.1, frame accuracy (Acc) increases by 0.4, and F1@{10,25,50} improves by 0.5, 0.6, and 0.5, respectively. To more intuitively demonstrate the improvement effects of our method on various datasets and to make a more direct comparison between our model’s results and those of other methods, we use visualized demonstrations with the 50Salads dataset as an example. Qualitative results are shown in Figure 3.

The superior performance of the proposed 2s-DAS framework is primarily attributed to the synergistic effect of the iterative diffusion process and multi-modal late fusion. Unlike single-pass predictive models, the diffusion module explicitly treats sequence prediction as a generative denoising process, which acts as an effective boundary refiner. By incorporating the importance sampling strategy during training, the model prioritizes key frames and learns the prior distribution of human actions robustly. Furthermore, the two-stream architecture captures highly complementary spatial-temporal cues: the Br-Prompt RGB stream provides rich static semantic context, while the I3D optical flow stream captures dynamic motion patterns. Consequently, 2s-DAS is most beneficial under conditions where actions exhibit distinct motion dynamics and complex transitions that rely heavily on global contextual reasoning. While 2s-DAS demonstrates strong numerical results, the performance gains are not equally distributed across all datasets. As shown in Table 1, the improvements on 50Salads and GTEA are substantial (e.g., increasing the Edit score by 3.0 and 3.8, respectively, compared to DiffAct). However, the gains on the Breakfast dataset are relatively marginal (edit score improvement of 1.1). This discrepancy stems from the inherent complexity of the data. The Breakfast dataset is significantly larger and more complex, featuring 48 distinct hierarchical actions across 1712 videos with extreme sequence lengths. Furthermore, as mentioned above, the Br-Prompt RGB features yielded poor representational performance on Breakfast, necessitating the use of I3D RGB features instead. This substitution inherently limited the multi-modal synergy compared to the optimal Br-Prompt and I3D Flow combination used for GTEA and 50Salads.

Considering computational cost, we evaluate our 2s-DAS against the single-stream baseline in terms of parameters, FLOPs, and inference speed, with the detailed results summarized in Table 2. Our 2s-DAS contains approximately 2.2 M parameters and 63.4 G FLOPs, compared to the 1.2 M parameters and 32.5 G FLOPs of the single-stream DiffAct baseline. While this increase in computational footprint is acceptable given the substantial performance boosts (e.g., an average 3% improvement across metrics on GTEA), the iterative sampling process of diffusion models combined with dual-stream decoding naturally affects the processing speed. As indicated in Table 2, 2s-DAS achieves an inference speed of 717 FPS, whereas the single-stream DiffAct operates at 1456 FPS. Although this represents a reduction in speed compared to the baseline, an inference speed of 717 FPS remains exceptionally high for the requirements of high-precision offline video analysis, such as surgical phase recognition, behavior logging, or skill assessment. Furthermore, Table 2 reveals that both models maintain a negligible peak GPU memory footprint (0.03 GB for 2s-DAS), demonstrating the high efficiency of our framework. Consequently, 2s-DAS is highly optimized for tasks where segmentation quality is the priority, providing a superior balance between structural accuracy and computational efficiency.

### 4.3. Ablation Study

We conduct ablation experiments on the 50Salads and GTEA datasets. We train the model separately using single Br-Prompt RGB and I3D FLOW features. The results show that both perform worse than our 2s-DAS, proving that the fusion of multiple modalities can provide more information and achieve better segmentation results. The relevant experimental results for the 50Salads and GTEA datasets are shown in Table 3. In addition, from the experimental results, we can see that the performance of Br-Prompt RGB features is better than I3D FLOW features, and its impact in the action segmentation task is greater. Therefore, when performing weighted averaging at the fusion layer, we assign a larger weight to the output information of the Br-Prompt RGB stream. We also conduct ablation experiments on the related weight parameters. Taking the split2 part of the 50Salads dataset as an example, the experimental results are shown in Table 4. From the table, it can be seen that when the parameters $\mu_{s}=0.6$ and $\mu_{t}=0.4$, the scores of the indicators are the highest. This is because the weights allow the model to retain more visual spatial information while adding additional optical flow dynamic information. As the RGB weight increases and the optical flow weight decreases, although the effect is still better than the evaluation result of a single RGB feature due to the addition of optical flow information, the performance gradually decreases as the supplementary optical flow information decreases.

**Figure 4:**
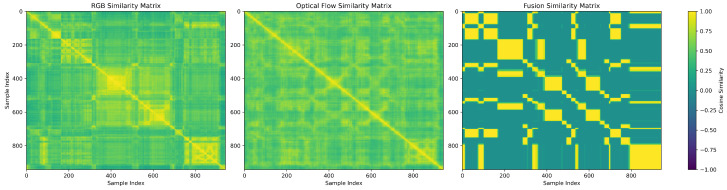
Visualization of modality interaction via cosine similarity matrices. From left to right: the similarity matrices of the RGB stream, the optical flow stream, and the fused representations. The late-fusion mechanism effectively suppresses the off-diagonal semantic noise from the RGB stream and sharpens the temporal boundaries of the optical flow stream, resulting in a highly discriminative block-diagonal structure.

To further investigate the underlying mechanism of this multi-modal interaction and explain the weight distributions, we conducted a feature-level similarity analysis. Figure 4 visualizes the cosine similarity matrices for the feature sequences extracted from the RGB stream, the optical flow stream, and the final fused representations. As shown in the figure, the RGB similarity matrix captures distinct semantic block structures but exhibits noticeable ambiguity and noise in off-diagonal regions, likely because different actions often share similar static environmental backgrounds. Conversely, the optical flow similarity matrix reflects continuous motion dynamics but lacks sharp, discriminative boundaries between distinct action transitions. Strikingly, the fused similarity matrix presents a highly discriminative and sharp block-diagonal structure. This explicitly demonstrates the synergistic interaction between the modalities: the late-fusion mechanism utilizes the dynamic motion cues from the optical flow to effectively suppress the static semantic noise from the RGB stream, while relying on the RGB stream to maintain strong semantic categorization. This interaction—using motion dynamics as a boundary sharpener for semantic representations—is the fundamental reason why our 2s-DAS significantly mitigates over-segmentation artifacts and achieves precise sequence modeling.

Table 5 shows more ablation experiments for our 2s-DAS (R,F) to compare the performance using each sampling and our random selection sampling. It shows that zero sampling is unsuitable and that our random selection outperforms each. Table 6 compares the results of our 2s-DAS (R,F) using three fusion strategies. Besides our late fusion, we also adopt early fusion (Br-Prompt RGB (R) and I3D flow (F) are concatenated before feeding) and mid-fusion (R and F are processed by two independent encoders and a shared denoising decoder). The superiority of late fusion is rooted in a critical theoretical characteristic of diffusion models: the prevention of cross-modal noise contamination. During the iterative reverse diffusion process, intermediate representations are highly chaotic. Since spatial-semantic features (RGB) and dynamic motion features (Flow) reside in fundamentally different manifold spaces, employing complex early or mid-fusion mechanisms inevitably causes the noise from one modality to leak into and corrupt the other. By isolating the two streams during the sensitive denoising phase, late fusion elegantly preserves the modality-specific structural integrity, merging only the purified predictive distributions. Consequently, our late fusion strategy significantly outperforms others in five metrics. It is worth noting that our method can also be easily applied to other backbone models. To prove this, we use the ASFormer [6] method as the backbone for our two-stream framework, and the new model is denoted as 2s-TAS [39]. Taking the 50Salads and Breakfast datasets as examples, our approach yields improvements on both datasets, with particularly significant enhancements on the 50Salads dataset, reaching levels close to state-of-the-art (SOTA) methods, as shown in Table 7. In the future, we can use more powerful backbone models to improve our experimental results.

### 4.4. Failure Case Analysis

**Figure 5:**
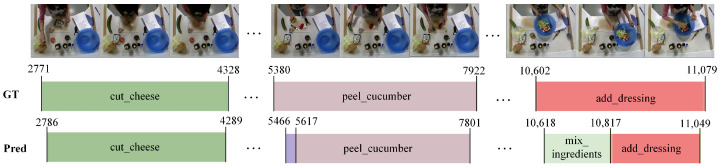
Qualitative visualization of typical failure cases on the 50Salads dataset. We highlight three primary error modes using red annotations: (**Left**) Boundary shifts, where the predicted action onset (e.g., frame 2786) lags behind the ground truth (frame 2771); (**Middle**) localized over-segmentation, where the model generates spurious, fragmented short actions (e.g., the purple segment before peeling) during ambiguous transition phases; and (**Right**) action misclassification, where the model incorrectly predicts “mix ingredients” instead of “add dressing” due to visually similar hand motions.

While the proposed 2s-DAS framework demonstrates superior quantitative performance and effectively mitigates overall over-segmentation, it is important to critically analyze its failure modes to understand its operational limitations. By inspecting the erroneous predictions on the highly challenging 50Salads dataset, we identify three primary error modes, as visualized in Figure 5. First, boundary shifts occasionally occur during continuous, fluid motion transitions (Figure 5, left). For instance, the predicted onset of the cut_cheese action slightly lags behind the ground truth. Predicting the exact frame where one fluid action ends and another begins remains a challenge, as these transition phases often lack distinct visual or kinematic breakpoints. Second, the model sometimes suffers from localized over-segmentation (Figure 5, middle). Although the diffusion-based decoder generally enforces strong sequence smoothness, highly ambiguous transition phases can cause high uncertainty between the modalities. This instability occasionally leads to the generation of spurious, fragmented short actions (e.g., the transient purple segment just before peel_cucumber). Finally, action misclassification remains a significant challenge under severe visual occlusion (Figure 5, right). In top-down datasets, when a subject’s hands obscure the interaction area, the Br-Prompt RGB stream may lose crucial semantic context regarding the manipulated objects. If the accompanying motion patterns captured by the optical flow stream are dynamically similar, the framework struggles to differentiate specific action classes (e.g., incorrectly predicting mix_ingredients instead of the ground truth add_dressing). Addressing these occlusion-heavy ambiguities with object-centric tracking remains an important direction for future research.

## 5. Conclusions

In this paper, we propose a two-stream diffusion framework named 2s-DAS for the task of temporal multi-modal action segmentation. To tackle the multi-modal input feature fusion and sequential modeling for segment-level prediction, we propose a multi-modal frame representation with I3D optical flow and Br-Prompt RGB features to enrich the feature representation, and we design the importance sampling block in the diffusion module to emphasize the key frames during the sequence segmentation, and a two-stream fusion mechanism that integrates multi-modal information from the optical flow stream and the RGB stream by a late fusion strategy, aiming to refine the prediction outputs and reduce the over-segmentation. Although this two-stream approach may require more computational resources and time, the 2s-DAS outperforms existing baseline models, achieving excellent results on three public benchmark datasets: GTEA, 50Salads, and Breakfast. One limitation of our current study is the domain diversity of the evaluation. Following the standard evaluation protocol in this field, our experiments are conducted on 50Salads, GTEA, and Breakfast, all of which focus on food preparation activities. While the proposed dual-stream architecture is designed for general temporal sequence modeling, its effectiveness in domains with fundamentally different visual and temporal dynamics—such as surgical phase recognition or industrial assembly—has not yet been verified. Therefore, applying and adapting our framework to these diverse real-world scenarios remains a key area for future work. In future work, we intend to extend the 2s-DAS framework to broader and more complex action understanding tasks. Specifically, we aim to evaluate the robustness of our diffusion-based multi-modal interaction mechanism on recent large-scale, fine-grained datasets, such as the Epic-Kitchens-100, Ego-Exo4D(CVPR2024), EgoSchema(ICLR2024), and Ego4D(CVPR2022) benchmarks for cross-view and long-form action recognition. benchmarks for cross-view and long-form action recognition. Adapting our framework to these massive, highly challenging datasets represents a promising direction toward generalized video understanding.

## Figures and Tables

**Table 1:** Comparison with the state-of-the-art on 50Salads, GTEA and the Breakfast dataset. R = RGB, F = FLOW. Bold and underlined denote the highest and the second-highest value in each column.

Method	50Salads			GTEA			Breakfast		
	F1@{10, 25, 50}	Edit	Acc	F1@{10, 25, 50}	Edit	Acc	F1@{10, 25, 50}	Edit	Acc
MS-TCN++ [18]	80.7 78.5 70.1	74.3	83.7	88.8 85.7 76.0	83.5	80.1	64.1 58.6 45.9	65.6	67.6
SSTDA [34]	83.0 81.5 73.8	75.8	83.2	90.0 89.1 78.0	86.2	79.8	75.0 69.1 55.2	73.7	70.2
ASRF [35]	84.9 83.5 77.3	79.3	84.5	89.4 87.8 79.8	83.7	77.3	74.3 68.9 56.1	72.4	67.6
ASFormer [6]	85.1 83.4 76.0	79.6	85.6	90.1 88.8 79.2	84.6	79.7	76.0 70.6 57.4	75.0	73.5
Br-Prompt [14]	89.2 87.8 81.3	83.8	88.1	94.1 92.0 83.0	91.6	81.2	-	-	-
UVAST [7]	89.1 87.6 81.7	83.9	87.4	92.7 91.3 81.0	92.1	80.2	76.9 71.5 58.0	77.1	69.7
LTContext [20]	89.4 87.7 82.0	83.2	87.7	-	-	-	77.6 72.6 60.1	77.0	74.2
DiffAct [9]	90.1 89.2 83.7	85.0	88.9	92.5 91.5 84.7	89.6	82.2	80.3 75.9 64.6	78.4	76.4
BaFormer [36]	89.3 88.4 83.9	84.2	**89.5**	92.0 91.3 83.5	88.7	**83.0**	79.2 74.9 63.2	77.3	76.6
ActFusion [37]	91.6 90.7 84.8	86.0	89.3	94.1 93.3 **86.9**	91.6	81.9	**81.0** 76.2 64.7	79.3	76.4
BIT [38]	-	-	-	**94.8** 92.8 82.6	92.6	82.0	80.6 75.8 64.7	79.0	75.5
**2s-DAS(R, F)**	**92.3** **91.7** **86.2**	**88.0**	**89.5**	**94.8** **94.0** 86.1	**93.4**	81.7	80.8 **76.5** **65.1**	**79.5**	**76.8**

**Table 2:** Comparison of computational complexity and inference efficiency. The best results are highlighted in bold.

Method	Params (M)	FLOPs (G)	GPU Mem (GB)	Speed (FPS)
DiffAct [9]	1.2	32.5	**0.06**	**1456**
**2s-DAS (Ours)**	**2.2**	**63.4**	0.03	717

**Table 3:** Ablation experiment results on GTEA and 50Salads. The best results are highlighted in bold.

Method	GTEA			50Salads		
	F1@{10, 25, 50}	Edit	Acc	F1@{10, 25, 50}	Edit	Acc
2s-DAS (F)	92.0 90.9 82.3	89.2	80.0	81.0 79.6 71.9	75.9	81.0
2s-DAS (R)	93.8 92.6 85.5	92.2	80.9	91.8 90.9 85.3	86.6	89.0
**2s-DAS (R, F)**	**94.8 94.0 86.1**	**93.4**	**81.7**	**92.3 91.7 86.2**	**88.0**	**89.5**

**Table 4:** The fusion weight experiments on 50Salads. The best results are highlighted in bold.

$\mu_{R}$	$\mu_{F}$	F1@{10, 25, 50}	Edit	Acc
0.8	0.2	91.6 91.1 87.4	85.9	90.1
**0.6 **	**0.4**	**93.2 92.2 89.1**	**88.8**	**90.7**
0.4	0.6	83.6 82.6 75.1	74.9	83.6
0.2	0.8	83.5 81.9 74.3	75.8	82.3

**Table 5:** Comparison of importance sampling strategies on GTEA. The best results are highlighted in bold.

Sampling Strategy	F1@{10, 25, 50}	Edit	Acc
Zero	36.4 27.6 13.2	62.7	29.4
Full	93.1 92.8 84.4	91.8	81.4
Boundary	93.5 93.3 84.2	89.9	81.4
Random segment	93.6 93.3 85.0	91.7	81.5
Random selection (Our)	**94.8** **94.0** **86.1**	**93.4**	**81.7**

**Table 6:** Comparison of multi-modal fusion strategies on GTEA. The best results are highlighted in bold.

Fusion Strategy	F1@{10, 25, 50}	Edit	Acc
Early fusion	93.5 92.2 85.6	90.9	81.6
Mid fusion	93.9 92.5 85.7	90.8	81.7
Late fusion (Our)	**94.8** **94.0** **86.1**	**93.4**	**81.7**

**Table 7:** Comparison of ASFormer and 2s-TAS. The best results are highlighted in bold.

Method	F1@{10, 25, 50}	Edit	Acc
ASFormer (50Salads)	85.1 83.4 76.0	79.6	85.6
2s-TAS (50Salads)	**91.0 89.6 85.1**	**84.9**	**90.1**
ASFormer (Breakfast)	76.0 70.6 57.4	75.0	73.5
2s-TAS (Breakfast)	**76.5 71.1 57.8**	**75.6**	**73.7**

## Data Availability

The data presented in this study are openly available in 2s-DAS at https://github.com/celicvml/2s-DAS (accessed on 10 May 2026).
